# Electrochemical Characterization and CO_2_ Reduction Reaction of a Family of Pyridazine-Bridged Dinuclear Mn(I) Carbonyl Complexes

**DOI:** 10.3390/molecules28031138

**Published:** 2023-01-23

**Authors:** Jacopo Isopi, Elsa Quartapelle Procopio, Lorenzo Veronese, Marco Malferrari, Giovanni Valenti, Monica Panigati, Francesco Paolucci, Massimo Marcaccio

**Affiliations:** 1 Dipartimento di Chimica “Giacomo Ciamician”, Università di Bologna, Via Selmi 2, 40126 Bologna, Italy; 2 Dipartimento di Chimica, Università di Milano, Via Golgi 19, 20133 Milano, Italy; 3 Consorzio INSTM, Via G. Giusti 9, 50121 Firenze, Italy

**Keywords:** electron transfer, cyclic voltammetry, CO_2_ reduction reaction, catalysis, manganese complexes

## Abstract

Three recently synthesized neutral dinuclear carbonyl manganese complexes with the pyridazine bridging ligand, of general formula [Mn_2_(μ-ER)_2_(CO)_6_(μ-pydz)] (pydz = pyridazine; E = O or S; R = methyl or phenyl), have been investigated by cyclic voltammetry in dimethylformamide and acetonitrile both under an inert argon atmosphere and in the presence of carbon dioxide. This family of Mn(I) compounds behaves interestingly at negative potentials in the presence of CO_2_. Based on this behavior, which is herein discussed, a rather efficient catalytic mechanism for the CO_2_ reduction reaction toward the generation of CO has been hypothesized.

## 1. Introduction

Owing to the fact that the applied potential to reduce CO_2_ into its radical anion CO_2_^•−^ has a rather negative value (E°(CO_2_/CO_2_^•−^)= −1.97 V versus SHE (standard hydrogen electrode)) in DMF [1], the catalysts used for the electrochemical CO_2_ reduction reaction (CO_2_RR) have been widely researched amongst molecular metal-based systems, as transition metals generally have multiple accessible redox states that facilitate multielectron chemistry. The vast majority of these catalysts employ a single metal center, [2,3,4,5,6,7,8] usually rhenium or manganese. Albeit structurally similar, Re- and Mn-based catalysts show different electrochemical behavior. While rhenium complexes have been studied for longer and are historically thought to be the most efficient in CO_2_RR, Chardon-Noblat, Deronzier, and co-workers [9] evidenced that Mn complexes (where the Mn center is typically in the Mn(I) oxidation state) can act as good CO_2_ reduction catalysts as well. Manganese-based complexes have become the focus of intense investigation in recent years as pre-catalysts for CO_2_ reduction, in part due to their high product selectivity for CO formation, but also because they are based on a cheaper and more earth-abundant metal, compared to their thoroughly investigated Re-based counterparts.

Mechanistic studies of rhenium-type catalysts have provided evidence for a concentration-dependent formation of dinuclear intermediates during catalysis [10,11,12,13,14,15]. However, only few examples have been related to catalysts with a rigid bridging ligand that links two rhenium or two manganese active sites in close proximity with a predictable intermetallic distance and orientation [16,17,18] in order to clearly highlight the contribution of the dinuclear pathway [19,20]. Indeed, cofacial dinuclear Re complexes, with a rigid backbone structure that prevents Re-Re bonding (leading to the deactivation of carbon dioxide reduction catalysis), showed a beneficial interaction between the two reaction sites, clearly outperforming non-cofacial or mononuclear complexes at a double concentration. In this framework, a new family of dinuclear tricarbonyl rhenium (I) complexes containing the 1,2-diazine bridging ligand and halide anions as ancillary ligands, has been reported, which is able to catalyze CO_2_ reduction with higher efficiency than that reported for the benchmark mononuclear complex Re(CO)_3_Cl-(bpy), confirming that the use of dinuclear complexes could be very promising in this field [21,22]. Although efficiencies are lower than those reported by Jurss and co-workers for similar dinuclear Re complexes (TOF = 15 s^−1^ for our complex vs. 35 s^−1^ for the complex reported by Jurss) [19] our work highlights a crucial role of the catalyst structure, in terms of the distance of metal centers and capability to facilitate CO_2_ insertion.

These complexes belong to the recently reported family of dinuclear Re(I) complexes containing a 1,2-diazine bridging ligand and two anionic ancillary ligands, such as halides [23,24], hydrides [25], alcohoxides, or chalcogenides [26,27], which can act as a bridge between two Re(CO)_3_ moieties.

On the basis of these results, we recently synthesized a new similar family of dinuclear manganese complexes containing 1,2-diazine as the bridging ligand and halides or chalcogenide as the ancillary ligands (structural determination and a thorough characterization of all the compounds investigated in this work is reported elsewhere) [28], which has the general formula [Mn_2_(μ-ER)_2_(CO)_6_(μ-pydz)] (pydz = pyridazine; E = O or S; R = methyl or phenyl). In addition to the rhenium counterpart, only two examples of dinuclear Mn complexes with two “Mn(CO)_3_” moieties connected by only one aromatic nitrogen bridging ligand have been reported to show higher performances [16,18]. It is worth noting that dinuclear manganese complexes (as those reported in this study)—containing not only the aromatic bridging ligand but also two anionic bridging ligands—have been investigated for the efficient CO release [29,30,31] and as electrocatalysts for proton reduction [32,33,34], but no examples have been reported for CO_2_ reduction reaction. 

**Scheme 1:**
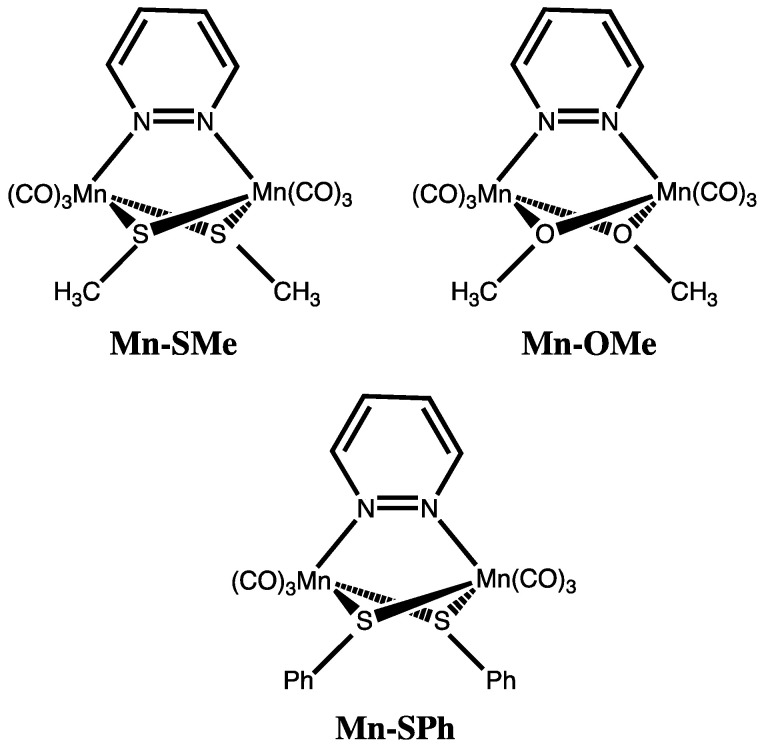
Molecular structure of the investigated Mn(I) dinuclear complexes.

In this work, for the first time, we studied the electrochemical behavior of this family of dinuclear Mn compounds (Scheme 1) in aprotic solvents and in the presence of CO_2_ to investigate its reduction reaction catalyzed by manganese-based complexes and to explore the beneficial effects on the catalytic performances of such a dinuclear geometry inspired by similar works concerning rhenium [21,22].

## 2. Results and Discussion

**Figure 1:**
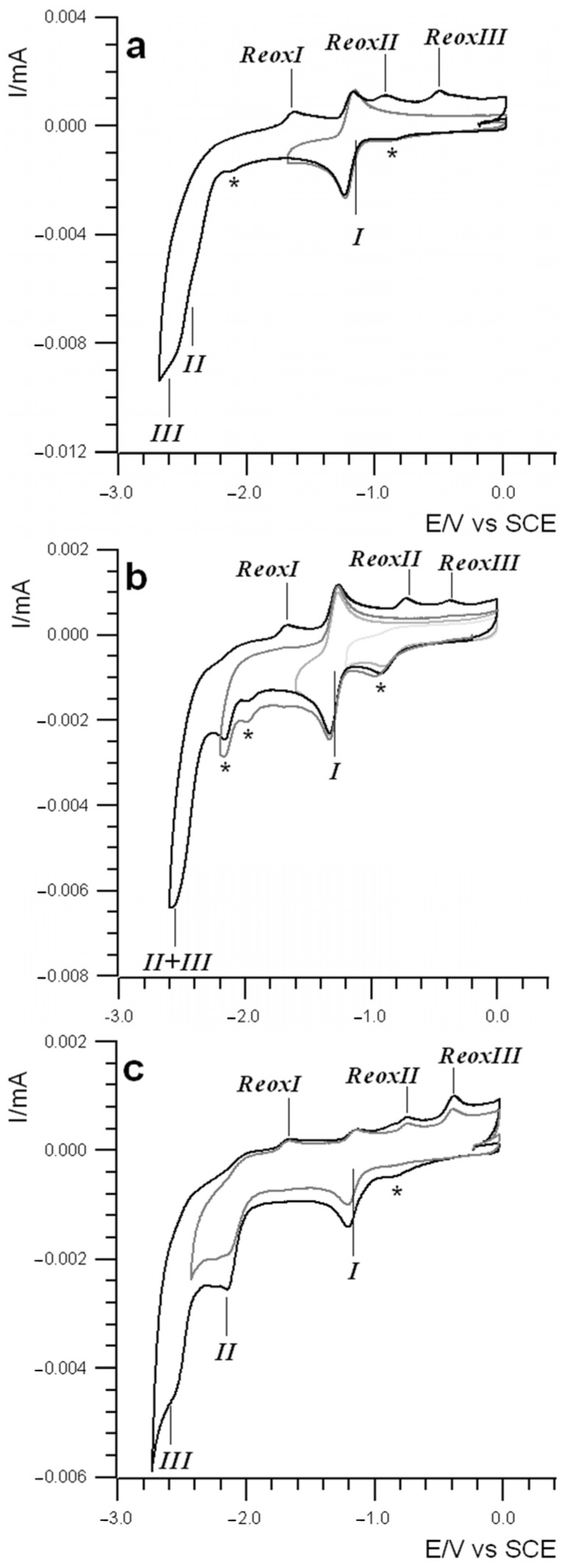
Cyclic voltammetry curves in Ar atmosphere of **Mn-SMe** (**a**), **Mn-OMe** (**b**), **Mn-SPh** (**c**). Solvent: DMF, scan rate: 0.4 V/s; T = 298 K. Reduction peaks indicated with * represent processes of unidentified decomposition products.

The electrochemical investigation of compounds **Mn-OMe**, **Mn-SMe**, and **Mn-SPh**, reported in Figure 1, was performed in DMF, which was chosen over acetonitrile (ACN) in order to avoid the higher coordinating properties of ACN molecules in the metal center. Electrolyte solutions of about 1 mM of each of the three studied complexes were prepared using dry DMF, and voltammetric measurements were taken in an inert atmosphere by Ar-saturating the solution. During the characterization procedures, the sample solutions were kept in a dark environment for the whole time, as Mn complexes are liable to photodegradation. All the systems were electrochemically characterized in a wide potential window by using a glassy carbon disk under the inert Ar atmosphere (Figure 1), before the introduction of CO_2_; the potentials of the most significant processes are reported in Table 1.

Herein, the voltammetric behavior of an Ar-saturated solution of the species **Mn-OMe**, **Mn-SMe,** and **Mn-SPh** was investigated in the negative potential range, as shown in Figure 1. For **Mn-SMe** and **Mn-OMe** (Figure 1a,b, respectively), the potential scan at negative values presented a completely reversible reduction with a half-wave potential E_1/2_***^I^*** at about −1.0 V (*vs* SCE) and centered on the diazine ligand (*vide infra*). The reversibility of this peak clearly indicates the good stability of these complexes towards processes involving ligand dissociation and the further formation of dimers containing Mn-Mn bonds, usually observed following the reduction of mononuclear tricarbonyl Mn complexes [9,35,36].

The first reduction was followed by a second voltammetric wave with a two-electron peak at about −2.3 V, which occurred just at the edge of the potential window allowed by the solvent. This last voltammetric peak was completely irreversible, and only a careful inspection of the voltammetric features highlighted the presence of two subsequent overlapping processes with potentials very closely spaced (labeled as II and III in Figure 1a). Moreover, on the backward potential scan, three small reoxidation processes were spawned with peak potentials reported in Table 1, thus suggesting a reaction mechanism of the molecule, following up the second reduction voltammetric peak (*vide infra* for a description of the mechanism). In the case of **Mn-OMe,** two other smaller reduction peaks were observed at −0.9 V and −2.2 V, respectively (indicated with asterisks in Figure 1b), that are related to the reduction of some decomposition products. In fact, although all the CV experiments were carefully carried out in dark conditions, the **Mn-OMe** derivative was highly photosensitive, even upon exposure to indirect sunlight, making the prevention of some partial decomposition impossible. The photostability of the complexes in different solvents has been investigated and fully reported elsewhere [28]. 

Figure 1c reports the electrochemical behavior of species **Mn-SPh,** for which the reversibility of the first peak was mostly lost. This is in agreement with the weaker donating power of the phenylsulfide anion with respect to the methylsulfide or methoxo anions. This led to the weakening of the Mn-S bond, resulting in a possible opening of the bridge bond, formed by the ancillary ligands, and thus a partial dissociation of the complex. As a consequence, the two-electron voltammetric wave beyond −2.0 V was split into two distinct one-electron processes.

**Figure 2:**
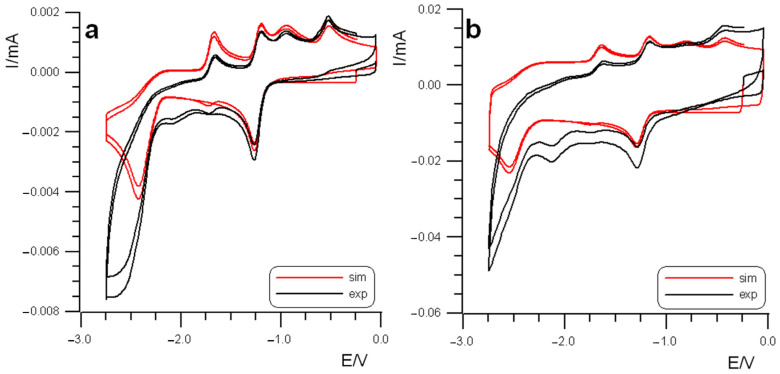
Simulated voltammetric curves (Antigona software version 1.0) juxtaposed on the original experimental data of **Mn-SMe**. Simulations parameters fitted using experimental CVs with scan rate 0.4 V/s (**a**) and 10.0 V/s (**b**). Ar atmosphere, TBAH/DMF electrolyte solution, working electrode: GC; reference electrode: SCE; T= 298 K.

To shed light on the behavior seen in the Ar-saturated DMF electrolyte solution, simulations were performed to understand a possible undergoing mechanism. In order to obtain a set of reliable and acceptable thermodynamic and kinetic parameters for the hypothesized mechanism (*vide infra*), the fit of the experimental voltammetric curves was obtained within a two-order-of-magnitude range of scan rates (between 0.4 and 10 V/s). Data for the **Mn-SMe** sample are reported below in Figure 2.

**Scheme 2:**
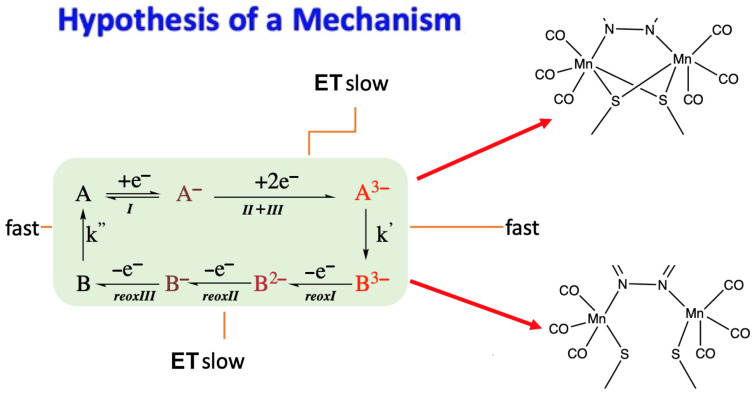
Simplified mechanism proposed to rationalize the experimental CV data obtained and to simulate the voltammetric curves of **Mn-SMe** in DMF solution. Double and single horizontal half-arrows represent electron transfers and full vertical arrows represent chemical reactions. The E° values for the various electron transfers and the kinetic constants of reactions are estimated from the simulation.

On the basis of the electrochemical and chemical behavior of the various species, the electrochemical mechanism that reasonably fits the experimental data is sketched below in Scheme 2, and the electrochemical and chemical simulation parameters are reported in Table S1 in the Supplementary Materials. In particular, it shows a reversible one-electron process followed by two irreversible closely spaced one-electron reductions. This second process led to three different oxidation peaks on the reverse anodic scan back to the initial potential.

The first reversible process can be reasonably attributed to the reduction of the aromatic diazine ring, not inducing any bond breaking. The irreversibility of the second voltammetric process (comprising two close one-electron transfers) indicates that the doubly and triply reduced molecule became unstable and that a follow-up chemical reaction has occurred. This can be associated with the double reduction of the two metal centers which most likely has a chemical impact involving the partial breaking of sulfur-Mn bonds between the ancillary bridging ligands and the Mn nuclei. 

In this respect, some preliminary molecular orbital DFT calculations on the **Mn-SMe** species were carried out, confirming that the first reduction is mostly centered on the diazine bridging ligand, while the subsequent two electron injections are mainly metal-centered. Moreover, while the molecular structure optimization at the M06/3-21G* level of the theory for the first reduced species **Mn-SMe** did not show any significant Mn-S bond elongation (only 0.02 Å), when the second electron was added, the optimization of the two-electron reduced the molecular structure, evidencing a rather large increase (0.22 Å) in one of the two Mn-SR bridging bonds for both the ancillary ligands. Such a 0.22 Å value is an undoubtedly a clear indication and theoretically supports the “opening” of the ancillary bridges (see Figure S1 in the Supplementary Materials).

From the new formed *isomeric* structure (right-bottom structure in Scheme 2), the re-oxidation to the original valence state was possible and the processes were again irreversible because the electron transfers were affected by chemical reactions. In fact, this last chemical process leads to re-establish the bonds involving the bridging sulfurs, thus revealing a square-scheme mechanism, as shown in Scheme 2. Thus, such a reaction mechanism can be involved in the interaction with CO_2_ and its reduction (*vide infra*). It should be noted that voltammetric curves recorded at lower scan rates (<0.4 V/s) show some degree of electrode passivation.

### Electrocatalysis

**Figure 3:**
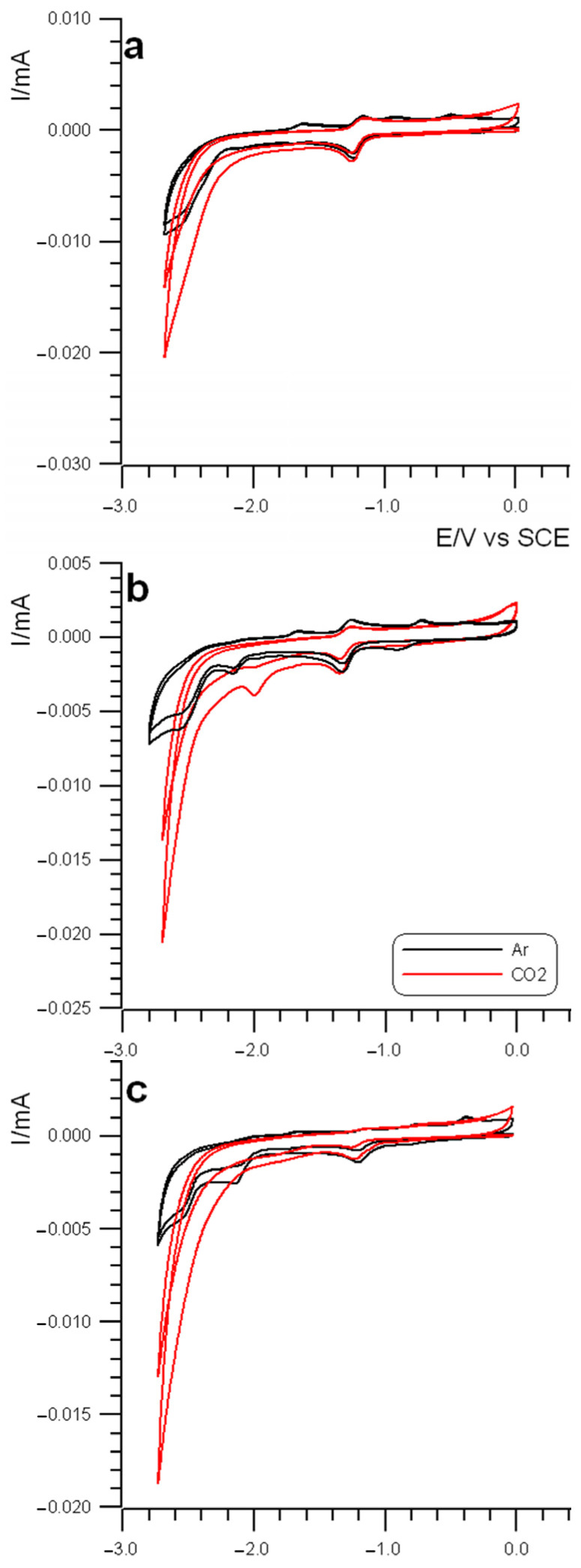
Cyclic voltammetry comparison between sample behavior in Ar (black) and in CO_2_ (red) saturated solution of **Mn-OMe** (**a**), **Mn-SMe** (**b**), **Mn-SPh** (**c**) in DMF. Working electrode: GC (7.85 × 10^−3^ cm^2^); reference electrode: SCE; scan rate: 0.4 V/s; T= 298 K.

After the preliminary voltammetric investigation in an inert atmosphere, the solution was saturated with CO_2_. The presence of CO_2_ led to a remarkable change in the voltammetric pattern with an increase in the current of the two-electron peak occurring at a potential more negative than −2.0 V for all four systems, thus indicating that the highly reduced transient species, electrogenerated at those potentials, are affected in some way by the presence of carbon dioxide. Such an increase in the current wave overlapped with the two-electron peak (i.e., processes ***II + III***) overwhelming it and revealing what looks like a solvent discharge, as shown in Figure 3 for all the Mn complexes. Moreover, it is worth noting the complete absence of the sequence of the three reoxidation processes on the reverse anodic scan, thus suggesting that the new process completely overturns the previously detected one in argon (Figure 3).

**Figure 4:**
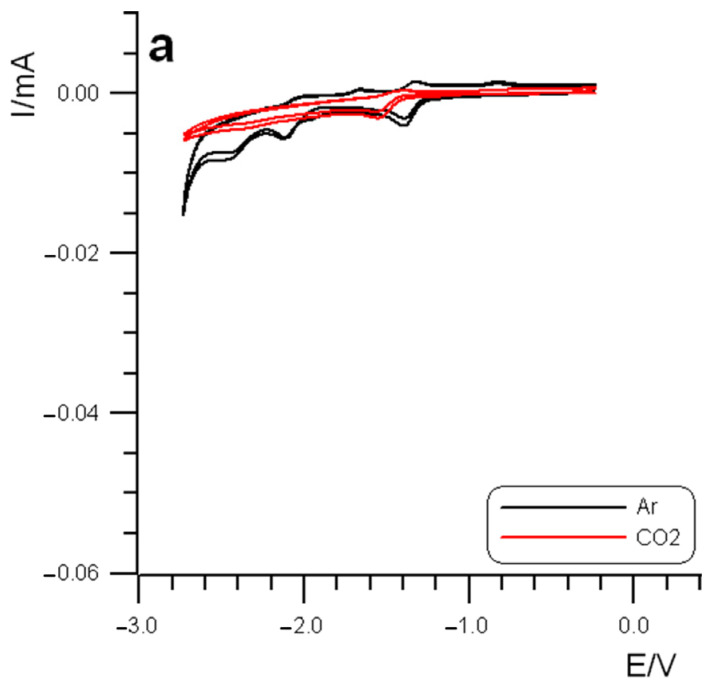

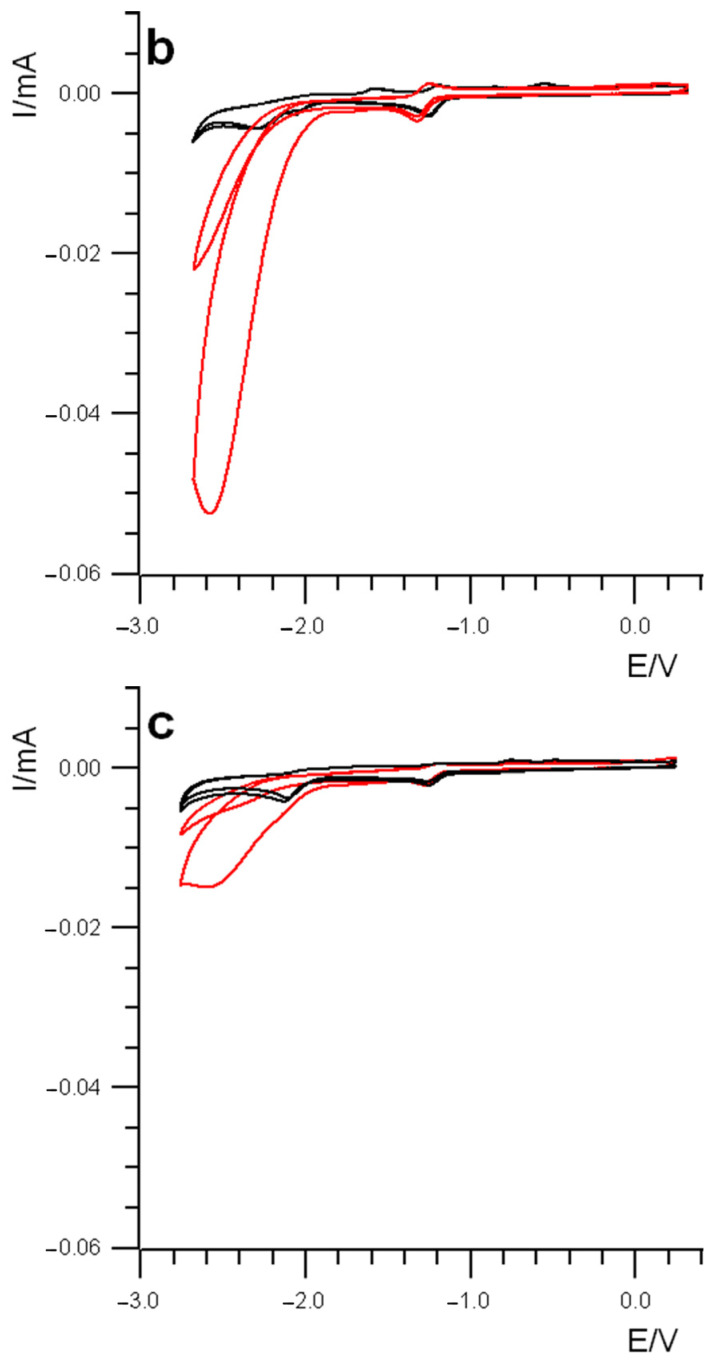
Comparison of the voltammetric behavior between **Mn-OMe** 0.6 mM (**a**), **Mn-SMe** 0.9 mM (**b**), and **Mn-SPh** 0.3 mM (**c**) in the TBAH/ACN electrolyte solution in the presence of Ar (black traces) and CO_2_ (red traces). Working electrode: GC disk (7.85 × 10^−3^ cm^2^); reference electrode: SCE; scan rate (υ): 0.4 V/s; T= 298 K.

A very similar voltammetric behavior was also observed in an acetonitrile solution. Figure 4 reports the CV curves of all the complexes in ACN in the presence of CO_2_, revealing a strong current increase at the second reduction peak potential, as observed in the DMF solution.

For the sake of comparison, a blank experiment without any of the manganese complexes in solution revealed that CO_2_ reduction was observed on a glassy carbon electrode only starting at −2.6 V, with a shift of ~0.4–0.5 V towards a more negative potential. 

Measurements showing a clear and efficient CO_2_RR, in particular for the dinuclear carbonyl Mn species **Mn-SMe** and **Mn-SPh**, allowed the turnover frequency (TOF) to be calculated for such systems. By comparing the relevant peak current in the presence of the substrate CO_2_ (i_cat_) and in its absence (i_p_), it was possible to calculate the TOF for a homogeneous catalyst (in the case of a reversible electron transfer reaction followed by a fast catalytic reaction) using the following equations:

(1) $$i_{cat}=n_{cat}FA\left[cat\right]{\left(Dk_{cat}{\left[Q\right]}^{y}\right)}^{1/2}$$

(2) $$i_{p}=0.4463n_{p}^{3/2}FA\left[cat\right]{\left(\frac{F}{RT}\right)}^{1/2}v^{1/2}D^{1/2}$$

(3) $$TOF=k_{cat}\left[Q\right]$$

where y is equal to 1, assuming pseudo-first-order kinetics, since the concentration of substrate Q (i.e., CO_2_) is large in comparison to the concentration of the catalyst [37]. Moreover, n_cat_ is the number of electrons required for the catalytic reaction (n_cat_ = 2 for the reduction of CO_2_ to CO), F is the Faraday’s constant, A is the surface area of the electrode, [cat] is the catalyst concentration, D is the diffusion constant of the catalytically active species, k_cat_ is the rate constant of the catalytic reaction, and [Q] is the substrate concentration. Equation (2) describes the peak current of a reversible electron transfer not affected by any follow-up reaction [38]. R is the universal gas constant, T is temperature, n_p_ is the number of electrons in the reversible non-catalytic reaction, and υ is the scan rate. Dividing Equation (1) by Equation (2) allows for the determination of i_cat_/i_p_ and the further calculation of the catalytic rate constant (k_cat_) and the turnover frequency (TOF). Solving i_cat_/i_p_ for $k_{cat}\left[Q\right]$ and combining with Equation (3), it is possible to obtain:

(4) $$TOF=k_{cat}\left[Q\right]=\frac{Fvn_{p}^{3}}{RT}{\left(\frac{0.4463}{n_{cat}}\right)}^{2}{\left(\frac{i_{cat}}{i_{p}}\right)}^{2}$$

In this equation, factor A is canceled out because the same electrode was used for the experiments under CO_2_ and Ar. The diffusion coefficient D is also canceled out because we assumed that the value of the catalytically active species does not change significantly under CO_2_ or Ar. By using experimental data from ACN solutions of **Mn-SMe** and **Mn-SPh** (Figure 4), the TOF values were calculated through Equation (4). 

Using the current peaks of the first voltammetric cycle (to exclude any eventual electrode passivation), the TOF values were 131 s^−1^ for **Mn-SMe** and 10 s^−1^ for **Mn-SPh**. Data from **Mn-SMe** and **Mn-SPh** show that they behaved much better, with a value above 7.2 s^−1^, as reported in previous works on manganese-based systems [39]. Scans conducted on **Mn-OMe** provide evidence that this Mn complex is inactive toward the CO2RR.

**Scheme 3:**
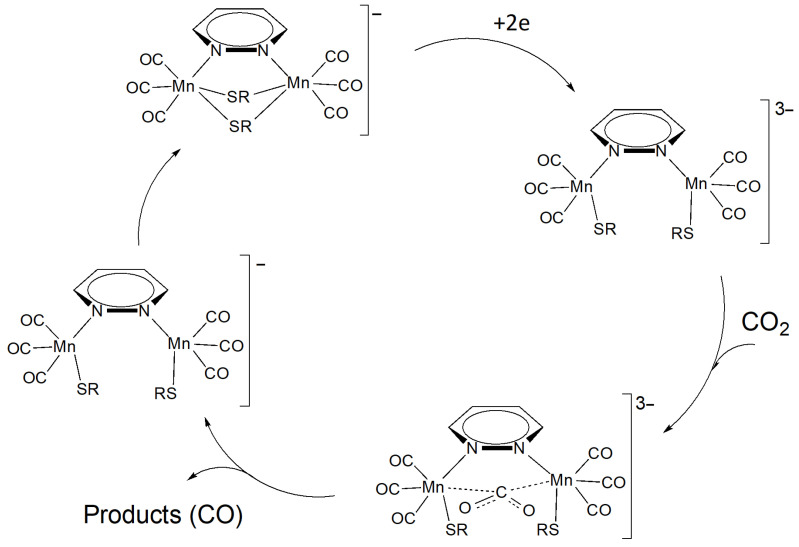
Sketch of the mechanism proposed for the catalysis of the CO_2_ reduction reaction by the **Mn-SMe** complex.

The notable large difference of activity between the O-based and S-based bridging moieties for the two systems **Mn-OMe** and **Mn-SMe**, respectively, is proof of the influence of the ancillary ligand on the catalytic performances, as reported for the analogous rhenium complexes [22]. In **Mn-SMe,** both Mn-S bond cleavages take place almost simultaneously, without the decomposition of the complex and/or the formation of an Mn-Mn direct bond, allowing both the metal centers to interact with CO_2_, as shown in Scheme 3. It is unlikely that two CO_2_ molecules have enough room to interact independently with both nuclei, but, as previously reported in the literature, a dinuclear reaction site can outperform two independent mononuclear ones, as demonstrated in Re-based systems [19]. 

However, in **Mn-SPh**, the two Mn-S bond cleavages can initiate a partial decomposition of the complex, due to the lower donating power of the phenyl derivative affording the splitting of the second voltammetric peak in two processes. Interestingly, the CO_2_RR peak takes place clearly when the species with one metal center, formed upon reduction, interacts with the substrate. On the other hand, the strong interaction of the OMe^−^ anion with the metal center, due to the hard nature of the ligand and its higher electronegativity, with respect to the SMe^−^ anion, showed to partially hamper the coordination of CO_2_, thus decreasing the efficiency.

## 3. Materials and Experimental Procedures 

Tetrabutylammonium hexafluoro-phosphate (TBAH) (99.0%) purchased from Sigma-Aldrich was used as electrolyte, and N,N-dimethylformamide (DMF) (99.5%) and acetonitrile (ACN) (99.5%) purchased from Sigma-Aldrich were used as solvents. Electrochemical investigations linked to cyclic voltammetry (CV) were carried out using a BioLogic SP-300 instrument and a custom-made electrochemical cell. A 1 mm diameter GC disk was used as the working electrode; the working electrode was polished with a 0.3 μm aluminium oxide (Buehler) slurry in distilled water on a felt pad. A Pt spiral wire acted as the counter electrode and an Ag wire was used as a pseudo-reference electrode that was checked to have a stable potential with a negligible drift within the time of the experiment comprising several potential sweeps in the electrolyte/solvent used. The potential scale was corrected vs the saturated calomel electrode (SCE) using the redox couple ferrocene/ferrocenium as an internal reference. The solvent, containing both DMF and ACN, was purified and dried by distillation and stored over activated 4 Å molecular sieves in a Schlenk flask. It was transferred through a cannula system into a custom-designed electrochemical cell containing the supporting electrolyte and the species under examination, immediately before performing the experiment [22]. The argon and carbon dioxide were initially bubbled through the electrolyte solution and as a blanket of gas at a pressure of 1 bar while measurements were performed.

All the E_½_ potentials have been directly obtained from cyclic voltammetric curves as averages of the cathodic and anodic peak potentials and by digital simulation in the case of non-Nernstian or overlapping processes. Digital simulations of the cyclic voltammetric curves were carried out by Antigona [40] or DigiSim 3.0 (Bioanalytical Systems, Inc. West Lafayette. IN, USA), utilizing a best-fitting procedure of the experimental curves recorded at different scan rates spanning over at least two orders of magnitude [41,42].

The chalcogenide-bridged dinuclear Mn carbonyl complexes herein investigated (see Scheme 1) were obtained through the one-pot synthesis “orthogonal bonding approach” [28], which was previously used for the synthesis of analogue rhenium dinuclear complexes [27] and different dinuclear manganese complexes [30]. The oxidative addition of E_2_R_2_ ligands (E = S and R = methyl or phenyl) to Mn_2_(CO)_10_ and the further addition of the pyridazine bridging ligand affords complexes labeled as **Mn-SMe** and **Mn-SPh** in good yields (about 60%). For the synthesis of the methoxo derivative **Mn-OMe**, methanol was used as the source of -OMe anions to obtain the corresponding managanese complex **Mn-OMe**. The structural determination of products and the thorough characterization of all compounds investigated in this work have been reported elsewhere [28].

## 4. Conclusions

We described and electrochemically characterized three manganese complexes with different bridging ancillary chalcogenide ligands as promising homogeneous CO_2_RR catalysts. As revealed by the analogous rhenium complexes, the catalysis efficiency, quantified using various methods outlined in the literature, confirms the strong correlation between the molecular structure and catalytic activity in CO_2_ reduction. The maximum TOF was observed for complex **Mn-SMe**, affording an impressive CO_2_RR activity with TOF values up of 131 s^−1^ that is much higher than those reported by previous works on manganese-based systems, reaching a maximum of 7.2 s^−1^. The three-order-of-magnitude difference between the two very similar systems also reveals interesting insight into the mechanism that takes place at the reaction site, showing the effect of the ancillary ligand on catalytic activity. We hypothesized a mechanism based on electrochemical findings, indicating that the reaction site for CO_2_RR is formed when the bond with the ancillary bridging ligand is cleaved so that the CO_2_ molecule can access the metal center. In particular, this feature is in agreement with the lower electronegativity of the sulfur with respect to oxygen, together with the higher donor power of methyl with respect to phenyl, which increases the electronic density of the Mn centers and improves the stability of the reduced species, thus increasing the catalytic activity.

## Figures and Tables

**Table 1:** Half-wave (E_1/2_) and peak redox potentials (vs SCE) of compounds recorded in TBAH/DMF solution at 25 °C.

*Species*	E_1/2_ or E_p_/V (Reduction)			E_p_/V (Re-Oxidation)		
	*I*	*II*	*III*	*ReoxI*	*ReoxII*	*ReoxIII*
**Mn-SMe**	−1.20	−2.44 ^a^;	−2.56 ^a^	−1.62 ^a^	−0.92 ^a^	−0.50 ^a^
**Mn-OMe**	−1.30	−2.52 ^a^	−2.60 ^a^	−1.68 ^a^	−0.72 ^a^	−0.36 ^a^
**Mn-SPh**	−1.18	−2.14 ^a^	2.58 ^a^	−1.68 ^a^	−0.74 ^a^	−0.38 ^a^

^a^ Peak potential.

## Data Availability

Not applicable.

## Supplementary Materials

The following supporting information can be downloaded at: https://www.mdpi.com/article/10.3390/molecules28031138/s1, Table S1: parameters for the simulation of the cyclic voltammetric curves; Figure S1: Theoretical optimized molecular structures of the complex **Mn-SMe** for the pristine species and the double reduced compound.

## Sample Availability

Samples of the compounds are available from the authors.
