# Supplementary material for: Electrochemical Characterization and CO2 Reduction Reaction of a Family of Pyridazine-Bridged Dinuclear Mn(I) Carbonyl Complexes
Source: Molecules. 2023 Jan 23;28(3):1138. doi: 10.3390/molecules28031138 (PMC9922005; doi:10.3390/molecules28031138)
Supplement: Supplementary file 1 [file molecules-28-01138-s001.zip › molecules-2153605-supplementary.pdf]

Supplementary Materials

Electrochemical Characterization and CO_2 Reduction Reaction of a family of Pyridazine-Bridged Dinuclear Mn(I) Carbonyl complexes.

Jacopo Isopi,[a] Elsa Quartapelle Procopio,[b] Lorenzo Veronese,[b] Marco Malferrari,[a] Giovanni Valenti,[a] Monica Panigati,[b,c] Francesco Paolucci,[a*] Massimo Marcaccio,[a*]

[a] *Dipartimento di Chimica "Giacomo Ciamician", Università di Bologna, via Selmi 2, 40126 Bologna, Italy;*

[b] *Dipartimento di Chimica, Università di Milano, via Golgi 19, 20133 Milano, Italy*

[c] *Consorzio INSTM, via G. Giusti 9, 50121, Firenze, Italy;*

e-mail addresses:

massimo.marcaccio@unibo.it

francesco.paolucci@unibo.it

Table S1. Electrochemical and chemical parameters used for the simulation of the cyclic voltammetric curves reported in Figure 2 through the mechanism sketched in Scheme 2.

Redox process ***or*** ***Chemical reaction***	***Label mechanism process***	***Kinetic parameters***
A / A^-	***I***	**k**$_{s0}$ = 0.1 cm/s; (alpha = 0.5)
A^- / A^{2-}	***II***	**k**$_{s1}$ = $7 \bullet 10^{-4}$ cm/s; (alpha = 0.45)
A^{2-} / A^{3-}	***III***	**k**$_{s2}$ = 10^4 cm/s; (alpha = 0.45)
A^{3-} <=> B^{3-}	**k'**	**k**$_{3a}$ = 10^4 s^{-1}; **k**$_{3r}$ = 10^{-4} s^{-1}
B^{2-} / B^{3-}	***ReoxI***	**k**$_{s4}$ = 0.1 cm/s; (alpha = 0.5)
B^- / B^{2-}	***ReoxII***	**k**$_{s5}$ = $3 \bullet 10^{-3}$ cm/s; (alpha = 0.65)
B / B^-	***ReoxIII***	**k**$_{s6}$ = 0.03 cm/s; (alpha = 0.5)
B <=> A	**k''**	**k**$_{7a}$ = 10^4 s^{-1}; **k**$_{7r}$ = 10^{-3} s^{-1}

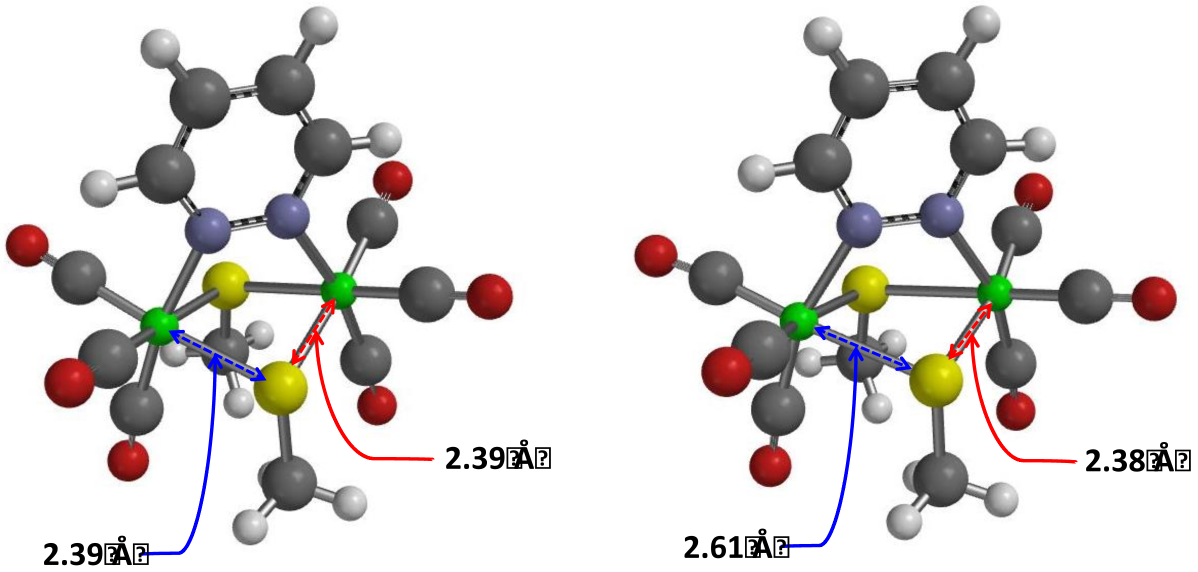

Mn-SMe
pris+ne

Mn-SMe
double reduced

Figure S2. Optimized molecular structures of the complex **Mn-SMe** at M06/3-21G* level of the theory for the pristine species (left) and the double reduced compound (right). The bond length of the ancillary bridging ligand with the two manganese centers are reported for the pristine and the double reduced species showing that one of the two bonds is elongated upon the 2-electron reduction, while the other remain unchanged. The same phenomenon occurs for the other ancillary bridging ligand.
